# The Impact of the COVID-19 Pandemic on Antibiotic Prescribing Trends in Outpatient Care: A Nationwide, Quasi-Experimental Approach

**DOI:** 10.3390/antibiotics10091040

**Published:** 2021-08-25

**Authors:** Tânia Magalhães Silva, Marta Estrela, Eva Rebelo Gomes, Maria Piñeiro-Lamas, Adolfo Figueiras, Fátima Roque, Maria Teresa Herdeiro

**Affiliations:** 1 Department of Medical Sciences, iBiMED—Institute of Biomedicine, University of Aveiro, 3810 Aveiro, Portugal; mestrela@ua.pt (M.E.); teresaherdeiro@ua.pt (M.T.H.); 2 Allergy and Clinical Immunology Service, University Hospital Center of Porto, 4099 Porto, Portugal; evamariasrg@gmx.com; 3 Consortium for Biomedical Research in Epidemiology and Public Health (CIBER Epidemiology and Public Health—CIBERESP), 15706 Santiago de Compostela, Spain; maria.pineiro@usc.es; 4 Health Research Institute of Santiago de Compostela (IDIS), University of Santiago de Compostela, 15706 Santiago de Compostela, Spain; 5 Research Unit for Inland Development, Guarda Polytechnic Institute (UDI-IPG), 6300 Guarda, Portugal; froque@ipg.pt; 6 Health Sciences Research Centre, University of Beira Interior (CICS-UBI), 6200 Covilhã, Portugal

**Keywords:** antibiotics, COVID-19, prescription, outpatient care, Portugal

## Abstract

Coronavirus disease 2019 (COVID-19) has spread globally and is currently having a damaging impact on nearly all countries in the world. The implementation of stringent measures to stop COVID-19 dissemination had an influence on healthcare services and associated procedures, possibly causing antibiotic consumption fluctuations. This paper aims to evaluate the immediate and long-term impact of the COVID-19 pandemic on antibiotic prescribing trends in outpatient care of the Portuguese public health sector, including in primary healthcare centers and hospitals, as well as on specific antibiotic groups known to be closely associated with increased resistance. Segmented regression analysis with interrupted time series data was used to analyze whether the COVID-19 pandemic had an impact in antibiotic prescribing tendencies at a national level. The outcomes from this quasi-experimental approach demonstrate that, at the beginning of the pandemic, a significant, immediate decrease in the overall antibiotic prescribing trends was noticed in the context of outpatient care in Portugal, followed by a statistically non-significant fall over the long term. The data also showed a significant reduction in the prescription of particular antibiotic classes (antibiotics from the Watch group, 3rd-generation cephalosporins, fluoroquinolones, and clarithromycin) upon COVID-19 emergence. These findings revealed an important disruption in antibiotics prescribing caused by the current public health emergency.

## 1. Introduction

The coronavirus disease 2019 (COVID-19) outbreak has quickly emerged as a public health emergency worldwide, with countries gathering all their efforts to combat this dangerous threat [1]. On 11 March 2020, the World Health Organization (WHO) officially characterized COVID-19 as a pandemic [2]. By 6 May 2021, more than 153 million of people were globally infected, with reports of 3.2 million deaths [3].

In Portugal, the declaration of the first state of emergency took place on 18 March 2020, with the government implementing a number of rigorous measures aiming to contain the virus transmission and COVID-19 dissemination, including the mandatory home confinement, which persisted until May 2020 [4], together with isolation, social distancing, and routine use of masks [5,6]. By 6 May 2021, there were approximately 838 thousand infections in Portugal and nearly 17 thousand deaths due to COVID-19 [7].

As a response to the quick escalation in the number of COVID-19 infections, a clinical guidance document for managing COVID-19 patients during all stages of the disease was published by the WHO [8]. Although the WHO’s recommendations for patients with mild or moderate COVID-19 are against the prescription of antibiotic therapy, unless there is a clinical suspicion of a bacterial infection or co-infection, this practice was shown to be common within hospitals [8,9,10,11,12]. Various studies have shown that some antibiotics were used to treat COVID-19 inpatients as a combination (e.g., azithromycin used in combination with other drugs due to its immunomodulatory effect [13,14]) or alternative therapies [15,16].

In fact, in 2019, an antibiotic classification list was released by the WHO entitled the 2019 Access, Watch, Reserve (AWaRe) classification of antibiotics for an improved evaluation and monitoring of use [17]. Using this classification is highly recommended by the WHO to support the development of antibiotic stewardship at the local, national, and global levels [17]. The Watch group includes 110 first- or second-choice antibiotics that display a higher resistance potential when compared with the Access group, and thus should be tightly monitored and restricted to the limited indications, as well as prioritized as major targets of stewardship programs.

The COVID-19 pandemic clearly had an impact in healthcare services and associated procedures, such as an increased number of hospitalizations and implementation of telemedicine in place of presential appointments [18,19,20,21]. There is a great concern about the impact imposed by the COVID-19 pandemic on the prescription of antibiotics and, eventually, on their associated resistance [22,23]. Two possible different outcomes concerning the prescription of antibiotics are to be considered: (i) an increment in prescriptions resulting from the empiric antibiotic prescribing through telemedicine [24]; or (ii) a decrease in prescriptions due to a lower number of medical appointments or due to the decline in respiratory infections [18,21].

The available data on the impact of the current pandemic on antibiotic use is still scarce [25]. The major goals of this study were to (1) evaluate the impact of the COVID-19 pandemic on the antibiotic prescribing tendencies in Portugal, in both primary healthcare centers and in outpatient care of hospitals, including emergency departments; and (2) assess the impact of the COVID-19 pandemic on specific antibiotic pharmacotherapeutic groups, particularly those closely related with increased antibiotic resistance, at a national level. Furthermore, the evaluation of the pandemic effects will be performed both at the immediate and long term.

## 2. Results

**Figure 1:**
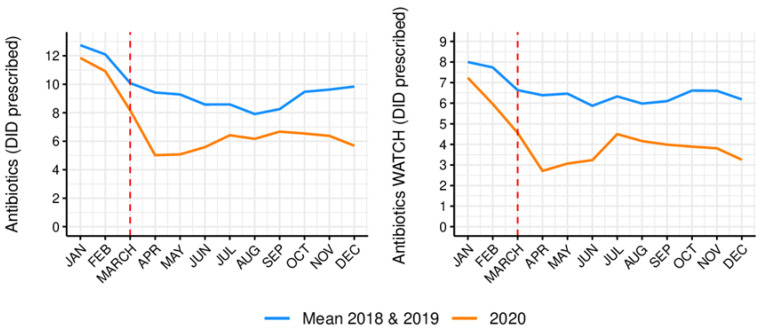
Changes in monthly antibiotic prescribing as DID in outpatient care, before and after COVID-19 emergence.

The comparison between the monthly prescription of antibiotics and of a group of antibiotics listed as Watch by the WHO, in the defined daily dose per 1000 inhabitants per day (DID) in outpatient care, before and after the emergence of the COVID-19 pandemic, is represented in Table 1. A high change in the percentage of antibiotic prescribing was observed, particularly from April 2020 onward, with decreases of around 50% when compared to the mean monthly prescription in 2018/2019 (Figure 1).

### 2.1. The Impact of the COVID-19 Pandemic on the Prescription of Antibiotics in Outpatient Care

**Figure 2:**
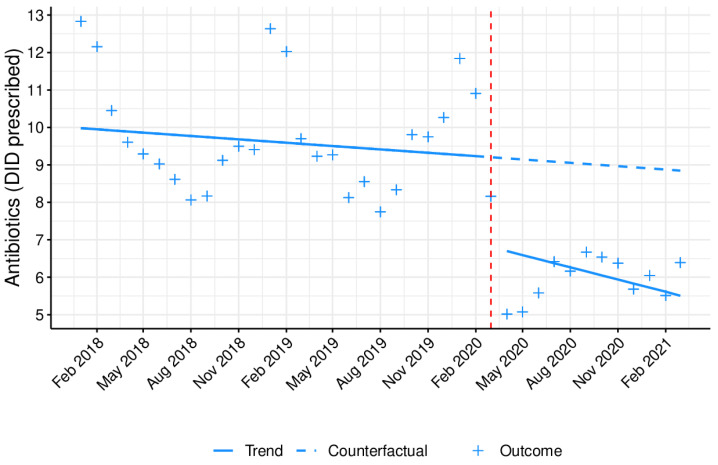
Monthly prescribing trend of antibiotics as DID prescribed in outpatient care. Blue line (−): tendency line, after model adjustment; blue dashed line: expected tendency line with no COVID-19 emergence; red dashed line: COVID-19 emergence (March 2020); **+**: antibiotic monthly prescription in DID.

Figure 2 illustrates the monthly antibiotic prescribing tendency between January 2018 and March 2021 for outpatients in primary healthcare and hospital centers. A high seasonality with increased prescribing peaks during the colder months was observed. Following the antibiotics trend evaluation, a segmented regression analysis was carried out, as displayed in Table 2. After seasonality adjustment, the overall antibiotic prescribing trend prior to the pandemic outbreak has not significantly changed (B = −0.009). Upon COVID-19 emergence, a significant (*p* < 0.05), sharp, and immediate decrease in antibiotics prescribing was observed in outpatient care (B = −1.6), followed by a small, non-significant (*p* = 0.83) rise (B = 0.011) over the long term.

### 2.2. The Impact of the COVID-19 Pandemic on the Prescription of Antibiotics Associated with Increased Antibiotic Resistance in Outpatient Care—Antibiotics from the WHO’s Watch Group

**Figure 3:**
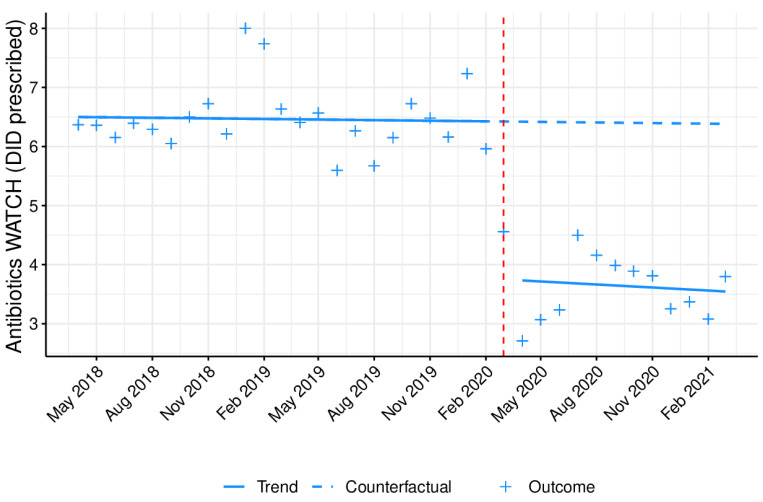
Monthly prescribing trend of antibiotics from the Watch WHO group as DID prescribed in outpatient care. Blue line (−): tendency line, after model adjustment; blue dashed line: expected tendency line with no COVID-19 emergence; red dashed line: COVID-19 emergence (March 2020); **+**: antibiotic monthly prescription in DID.

Figure 3 displays the monthly prescribing trend in DID in outpatient care of a group of antibiotics listed as Watch by the WHO, from April 2018 to March 2021, as the data from January to March 2018 were not available, and thus could not be included. A significant (*p* < 0.05), immediate reduction in the prescription of this specific antibiotic group was mainly detected at a national level after COVID-19 emergence (Table 3), with no statistically significant long term effect being noticed.

**Figure 4:**
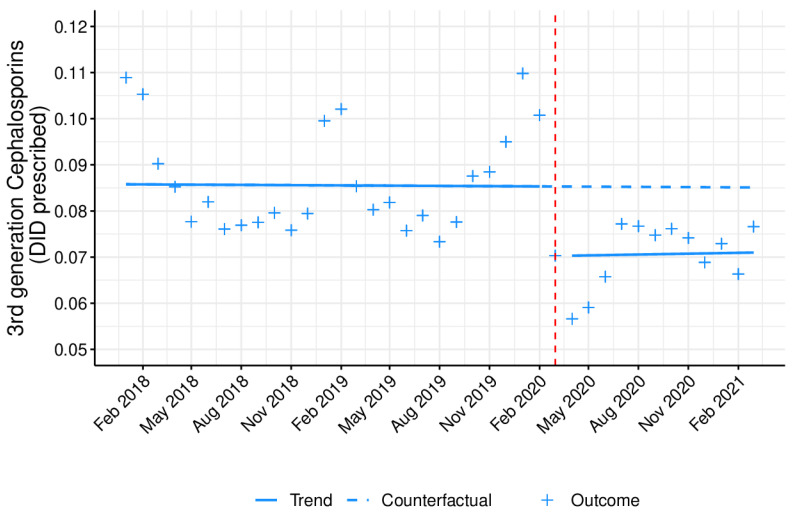
Monthly prescribing trend in 3rd-generation cephalosporins as DID prescribed in outpatient care. Blue line (−): tendency line, after model adjustment; blue dashed line: expected tendency line with no COVID-19 emergence; red dashed line: COVID-19 emergence (March 2020); **+**: antibiotic monthly prescription in DID.

**Figure 5:**
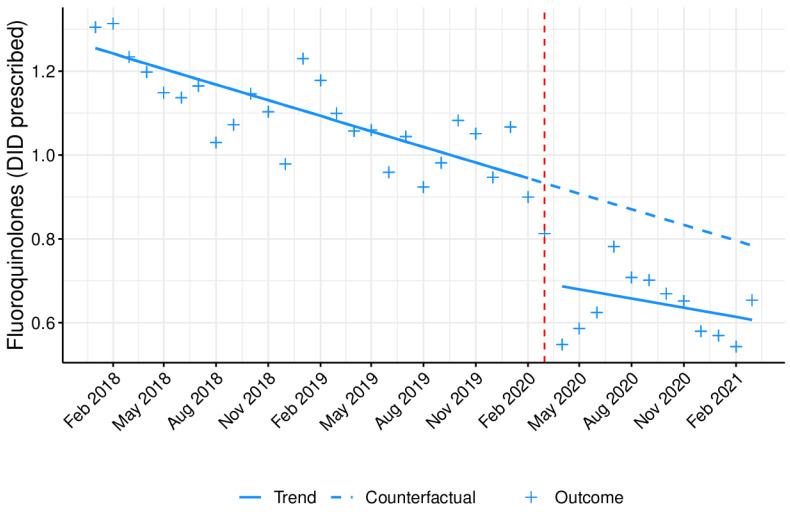
Monthly prescribing trend of fluoroquinolones as DID prescribed in outpatient care. Blue line (−): tendency line, after model adjustment; blue dashed line: expected tendency line with no COVID-19 emergence; red dashed line: COVID-19 emergence (March 2020); **+**: antibiotic monthly prescription in DID.

**Figure 6:**
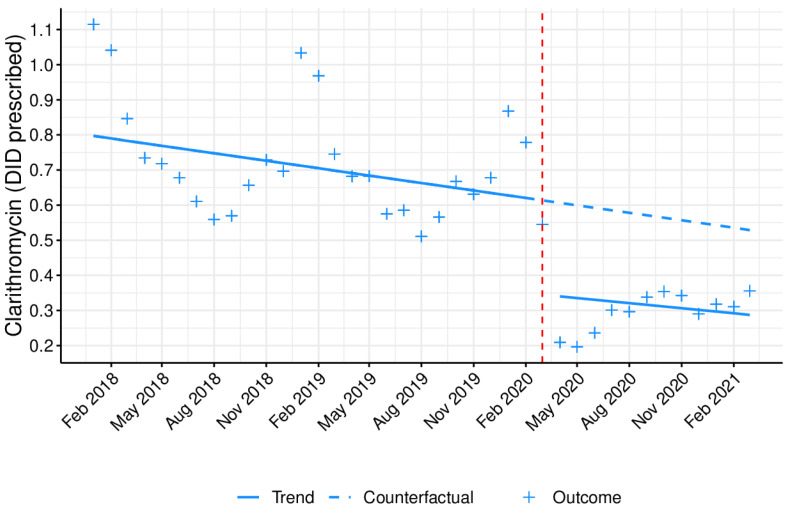
Monthly prescribing trend of clarithromycin as DID prescribed in outpatient care. Blue line (−): tendency line, after model adjustment; blue dashed line: expected tendency line with no COVID-19 emergence; red dashed line: COVID-19 emergence (March 2020); **+**: antibiotic monthly prescription in DID.

Figure 4, Figure 5 and Figure 6 represents the monthly prescribing tendency in DID, during the abovementioned period, of three antibiotic pharmacotherapeutic classes known to be closely associated with increased resistance and used against pathogens listed as Critical Priority and High Priority by the OMS and clarithromycin. Overall, the prescription of 3rd-generation cephalosporins (Figure 4) was shown to be residual and somewhat constant, while the prescribing trend of fluoroquinolones (Figure 5) and clarithromycin (Figure 6) was higher in the colder months, peaking in January.

**Figure 7:**
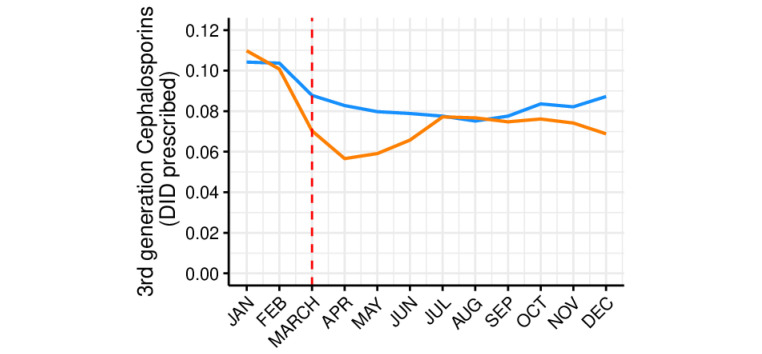

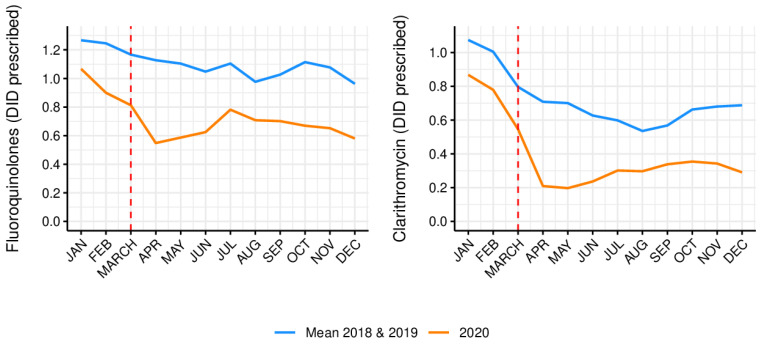
Changes in the monthly prescription of several antibiotic groups and clarithromycin as DID in outpatient care, before and after COVID-19 emergence.

The prescription of 3rd-generation cephalosporins, fluoroquinolones, and clarithromycin was significantly (*p* < 0.05) affected by the COVID-19 pandemic, with a decrease in the immediate but no statistically significant changes in the long term (Table 2). These data were reinforced by an overall decrease noticed in the monthly antibiotic prescribing percentage (Table S1 and Figure 7), when comparing the two previous years with 2020, particularly after March (when the first state of emergence was declared in Portugal due to the COVID-19 pandemic).

### 2.3. Sensitivity Analysis: The Effect of the COVID-19 Pandemic on the Prescription of Oral Antidiabetics

Overall, the prescription of oral antidiabetics (Supplementary Materials Figure S1) was generally constant from 2018 to 2021, with a few small falls occurring particularly in August and December. The segmented regression analysis data (Table S2) demonstrated that the COVID-19 pandemic did not have any immediate significant impact in the prescription of oral antidiabetics in outpatient care. Moreover, no statistically significant changes were observed in the long term (i.e., during the months following the pandemic escalation).

## 3. Discussion

### 3.1. Most Relevant Results of the Study

Worldwide, there is great concern about how the COVID-19 pandemic may affect healthcare systems, associated procedures, and prescriptions. Inadequate antibiotic prescribing may potentially contribute to the development and spreading of antibiotic resistances [9,25,26,27]. This quasi-experimental national approach carried out in the context of outpatient care in Portugal indicates that the current pandemic is associated with an immediate decrease in the overall antibiotic prescribing following COVID-19 emergence, but with no statistically significant reduction in the long term. The same effects were also observed within the antibiotics from the Watch group, 3rd-generation cephalosporins, fluoroquinolones, and clarithromycin.

### 3.2. Interpretation of the Main Results

The significant decrease noticed in antibiotic prescribing trends due to the COVID-19 pandemic may be explained by multiple factors. The mandatory home confinement, social distancing, isolation, and routine use of masks [5,6,12] implemented by the national government following the declaration of the state of emergency, together with improved hand hygiene practices [6,28], may have resulted in an overall decrease in transmissible infections, such as the very common respiratory infections, a decline in contagions, and, consequently, in antibiotic prescribing. With most of the people working from home, the exposure risk to bacterial agents was much lower when compared with the homologous period from previous years [29]. Another possible explanation is related with the cancellation of countless presential doctor appointments during this period [18,21], which could also have led to a decrease in antibiotic prescribing.

Nevertheless, the decline in antibiotic prescribing potentially caused by the abovementioned factors may as well be counterbalanced. The literature [9,30,31] has shown that over the first months of the pandemic, when COVID-19 test results were taking longer, antibiotic therapy was commonly prescribed to many patients before diagnosis—including COVID-19 inpatients—as these individuals could be potentially co-infected with bacteria, in addition to the enhanced risk of healthcare-associated infections together with the transmission of multidrug-resistant organisms resultant from hospital admissions [9,12,30,31]. Interestingly, the statistically significant immediate decrease observed in antibiotic prescribing in outpatient care may suggest that the COVID-19 pandemic was responsible for the occurrence of changes in the usual antibiotic prescribing trends in this context.

The period between April and May 2020 was, by far, the one displaying the lowest levels of antibiotic prescribing, with less than half when comparing to the homologous period from the two previous years. In fact, during these first pandemic months (particularly for April and May 2020) we have obtained falls in monthly prescription higher than 45% in antibiotics (J01) and over 50% in the antibiotics from the Watch group. Afterwards, with the suppression of the state of emergency and resultant home deconfinement, antibiotic consumption started to rise again, even though it did not reach the values from former years. All this valid evidence should be considered to help explain the statistically significant differences encountered in antibiotic prescribing tendencies in outpatient care upon COVID-19 emergence.

### 3.3. Secondary Outcomes

As a secondary outcome, this quasi-experimental approach demonstrated an immediate, significant impact of the COVID-19 pandemic on the prescription of specific antibiotics or antibiotic pharmacotherapeutic classes with higher therapeutic value, which are the most relevant for public health in terms of resistance. The group of antibiotics listed as Watch by the WHO comprises antibiotic classes displaying a higher risk of bacterial resistance and contains several of the highest priority agents among the critically important antimicrobials for human medicine, and should thus be tailored and prescribed sparingly to very specific settings [17]. In fact, among the 110 antibiotics from this group, 11 are included as first- or second-choice empiric treatment options for a limited number of infectious disorders [17]. Although the prescription of these antibiotics was shown to be very high in outpatient care in Portugal, a significant, immediate decrease was still observed upon COVID-19 emergence, possibly due to the restrictive confinement and increased hygiene measures adopted during the pandemic and the cancellation of doctor appointments. Moreover, this decrease in the primary care Watch antibiotic group prescribing has also been observed in another country [32]. With regard to the prescription of two of the antibiotic classes evaluated (3rd-generation cephalosporins and fluoroquinolones), together with clarithromycin, a statistically significant immediate decline was noticed, which favors a decrease in antibiotic resistance [33,34]. Nonetheless, this effect did not last long, as no significant changes were detected in the long term for any of the abovementioned drugs. It is also important to highlight that most of the prescriptions of 3rd-generation cephalosporins originate from outpatient hospitals, clinics, and emergency departments.

### 3.4. Comparison with Other Studies

This study seems to be in agreement with the official report by INFARMED, I.P., with Portugal registering a noticeable drop in antibiotics use between January and September 2020, when comparing with the homologous period from 2019 [35]. During this time, antibiotic dispensing in community pharmacies suffered a 20% fall [35]. Consistent with our data, after the “lockdown” in Scotland, a national evaluation of the impact of the COVID-19 pandemic on the community use of antibiotics revealed a reduction in the prescription of antibiotics commonly used for the treatment of respiratory infections, when compared to the same period from 2019 [36]. According to recent data from the European Center for Disease Prevention and Control (ECDC), Portugal fell below the European average regarding antibiotic consumption in 2019, both at primary healthcare centers (17.9 DID) and hospitals (1.4 DID) [37]. Overall, over the last 10 years, a slightly decreasing tendency was observed in national antibiotic consumption, with β-lactam antibacterials—penicillins (ATC group J01C)—followed by macrolides, lincosamides, and streptogramins (ATC group J01F) being the antibiotic classes with the highest average consumption [37]. In this nationwide study, the average for the overall antibiotic prescribing in outpatient care was of 9.69 DID in 2018, 9.62 DID in 2019, and 7.04 DID in 2020, thus highlighting the visible impact imposed by the COVID-19 pandemic on prescribing trends. This fall was shown to be higher in the prescription of fluoroquinolones (1.15 vs. 1.05 vs. 0.72 DID, respectively, in 2018, 2019, and 2020) and clarithromycin (0.75 vs. 0.69 vs. 0.40 DID, respectively, in 2018, 2019, and 2020) in outpatient care, including healthcare centers and hospitals.

### 3.5. Strengths and Weaknesses of the Study

We think that the most important strength of the current study is the methodology applied. The ITS design based on a segmented regression approach is the strongest, quasi-experimental design that enables the evaluation of the overall impact imposed by the COVID-19 pandemic in antibiotic prescribing trends [12,38,39]. Moreover, this study took into consideration the seasonality adjustment for data analysis, and we used, as a control, the prescription of an antidiabetic drug group used for chronic conditions, which was shown to be unaffected by the emergence and prolongation of the COVID-19 pandemic, contrary to the antibiotic prescribing trends.

Nonetheless, this study also presents a few limitations. The model applied to this study assumed a linear trend in the outcome within each segment and this assumption of linearity may only hold over short intervals. Additionally, changes may follow non-linear patterns and the segmented regression approach aggregated monthly antibiotic prescribing data, which may introduce bias due to monthly changes and inability to analyze individual-level characteristics (such as the pandemic impact on specific days, including, for instance, the days when the state of emergency was declared and abolished). Another limitation of this study is associated with the data source, as the results here displayed were only obtained from outpatient care of the public health sector, and not from the private sector, and may thus not reflect the real consumption of the antibiotics within the entire population. Due to the high variability in the application of procedures for COVID-19 patients and local restrictions, the results obtained in this study may not be extrapolated to other settings or locations, and thus more studies are needed in diverse additional contexts.

## 4. Materials and Methods

### 4.1. Setting

Portugal has a population of around 10.3 million inhabitants [40] that are unevenly distributed, with a high density in the metropolitan areas of Lisbon and Oporto. The Portuguese National Health System (NHS) was created in 1979, a universal tax-financed system tendentially free-of-charge covering all residents in Portugal [41]. The Portuguese health system presently comprises three co-existing and overlapping systems (~25%), namely, the NHS, the health subsystems (special public and private insurance schemes for certain professions or companies), and private voluntary health insurance [41]. The healthcare delivery system in Portugal consists of a network of public and private healthcare providers [41]. In Portugal, the reimbursement of medicines is performed according to four categories of NHS coinsurance [41]. Antibiotics are partially financed by the NHS and the co-payment rate attributed to the patients corresponds to 69% of the total price, under the general regimen [42].

### 4.2. Study Drugs

The drug class targeted in this paper was the antibacterials for systemic use (Anatomical Therapeutic Chemical (ATC) code J01) [43], from now on referred to as antibiotics.

As different antibiotic classes are usually associated with a different risk of resistance development, we also analyzed data concerning particular groups of antibiotics, such as the Watch group from the WHO’s AWaRe classification. [17]. We were able to retrieve the monthly prescribing data of 67 of these antibiotics, including azithromycin, ceftazidime, ciprofloxacin, meropenem, oxytetracycline, rifamycin, streptomycin, and vancomycin, among others. The remaining 43 are not prescribed in outpatient care and thus were not in the database drug list. Furthermore, the WHO Priority Pathogens List published in 2017 reports the prioritization of pathogens to guide research and development of new antibiotics in the form of a priority list of antibiotic-resistant bacteria, apart from *Mycobacterium tuberculosis* [44]. This list contains pathogens of critical, high, and medium priority, together with the associated resistant antibiotics. For this paper, we have extracted the monthly prescribing data of antibiotic classes resistant to the critical and high priority pathogens, including third-generation cephalosporins, fluoroquinolones, and the antibiotic clarithromycin. The antibiotics methicillin and vancomycin, and the carbapenems class were not used as they are either not prescribed or prescribed in very low levels in the context of outpatient care.

### 4.3. Study Design and Data Source

We conducted a natural, before–after, quasi-experimental approach to evaluate changes in the antibiotic prescribing trends associated with the declaration of the state of emergency in Portugal on 11 March 2020, by using a controlled interrupted time series (ITS) design. The time-series study was based on monthly antibiotic use data from January 2018 to March 2021, except the antibiotics from the Watch group (April 2018 to March 2021). This design allows for effects to be estimated by controlling for the baseline level and trends [45], with the longitudinal nature of the data providing a special robustness [46].

All data concerning drug prescribing were retrieved from the official System of Information and Monitoring of the Portuguese NHS (SIM@SNS) public-access platform [47], which was developed by the shared services of the Health Ministry (Serviços Partilhados do Ministério da Saúde—SPMS). This study only comprised healthcare institutions from the public sector associated to the Ministry of Health and included 44 hospitals. Some healthcare institutions were excluded from the prescribing data extraction as they are very specialized for certain health conditions, in addition to not being associated with the Health Ministry. These exclusions also included administrative institutions of the National Health Service, addiction rehabilitation centers, as well as the Armed Forces hospitals, and both military and prison hospitals, and have not caused any type of bias, as (1) they represent a very reduced percentage of the total number of prescriptions from the entire country; and (2) they affect both the pre- and post-pandemic period.

The antibiotic prescribing data retrieved comprised the defined daily doses (DDDs) of drugs prescribed between January 2018 and March 2021 in primary healthcare centers and hospital outpatient visits for medical specialty appointments and emergency departments, being extracted as dependent variables. Accordingly, all drug prescriptions provided by a doctor due to a hospitalization (involving at least an overnight hospital stay) and administered in the context of hospital care were not included in this study, due to the unavailability of data from the public sector. Afterwards, each monthly prescribed DDDs collected were converted into monthly prescribed DID, by using 10,295,909 as the 2019 annual estimate of the resident population in Portugal [48].

### 4.4. Statistical Analysis

Interrupted time series (ITS) has shown to be a very useful method to assess the impact of public health interventions [38,46]. An ITS analysis model based on a segmented regression approach for each independent variable was designed to analyze the differences between the monthly prescribed DID of antibiotics, with particular interest in those observed between March 2020 and March 2021. To detect variations in the prescription of some antibiotics, it is required to obtain monthly prescription comparisons with data from previous years [12].

Values were considered significant in any case where *p* < 0.05. The independent variables were defined as time in months (t: 1, 2, 3…, 39, except for the Watch group antibiotics, which goes only until 36); a binary variable (COVID), taking values of 0 before March 2020, and 1 from March 2020 onwards, corresponding to the time in which the effect of the pandemic is measured, and would thus show the immediate change; and a variable for the time elapsed since the declaration of the pandemic (post-COVID time), which took the value of 0 before and the values of 1, 2, 3…, corresponding to the months from March 2020 to March 2021 (long-term effect). To identify seasonal changes in antibiotic prescribing, the X-13ARIMA-SEATS procedure was applied [49]. This method is an adaptation of the US Bureau of the Census X-13–Auto-Regressive Integrated Moving Average (ARIMA) model that produces a seasonally-adjusted time series. For the Watch group antibiotics, as the monthly prescribing data available were not enough to apply the aforementioned procedure, the ITS analysis performed also took into consideration the independent temporal variable regarded as seasonality, but in this case assuming different values according to the winter (October until March) and summer months (April until September). Autoregressive terms were introduced into the models aiming to reduce any potential autocorrelation. This autocorrelation was controlled by introducing up to two lags of the response that were used only when *p* < 0.1.

All analyses were performed using the free R statistical software environment (version 4.0.5) [50].

### 4.5. Sensitivity Analysis

In order to assess whether the changes observed in antibiotic consumption were due to changes in the patterns of antibiotic prescribing or to global changes in the prescription and number of appointments, a sensitivity analysis was carried out using the oral antidiabetics therapeutic group (8.4.2) as a control group, as medicines used for chronic conditions should not be affected by the COVID-19 pandemic.

## 5. Conclusions

To the best of our knowledge, this is the first study highlighting the significant impact caused by the COVID-19 pandemic and related population-imposed measures on the antibiotic prescribing trends in outpatient care, from a nationwide point of view. At this unprecedented time, this countrywide study performed in Portugal indicates that the pandemic has reduced the global antibiotic prescribing in the immediate term in outpatient care. Nevertheless, several measures should be adopted by regulators and policy makers to monitor and reduce the use of the antibiotics from the Watch group in the public sector, as these display a higher resistance potential when comparing to the Access group of antibiotics. Additionally, we also believe that the availability of data from both primary healthcare centers and hospitals, not only in outpatient care, is very important to monitor the overall antibiotic consumption.

## Figures and Tables

**Table 1:** Monthly comparison analysis of antibiotics prescribing in outpatient care, before and after COVID-19 appearance.

		2018	2019	Mean 2018 and 2019	2020	Change in Percentage
**Antibiotic Prescribing (DID)**	JANUARY	12.8	12.6	12.7	11.8	−7.0
	FEBRUARY	12.2	12.0	12.1	10.9	−9.8
	MARCH	10.5	9.7	10.1	8.2	−19.0
	APRIL	9.6	9.2	9.4	5.0	−46.7
	MAY	9.3	9.3	9.3	5.1	−45.3
	JUNE	9.0	8.1	8.6	5.6	−34.9
	JULY	8.6	8.6	8.6	6.4	−25.2
	AUGUST	8.1	7.8	7.9	6.2	−22.0
	SEPTEMBER	8.2	8.3	8.3	6.7	−19.2
	OCTOBER	9.1	9.8	9.5	6.5	−30.9
	NOVEMBER	9.5	9.8	9.6	6.4	−33.8
	DECEMBER	9.4	10.3	9.8	5.7	−42.2
**WATCH Antibiotics Group Prescribing (DID)**	JANUARY *	-	8.0	8.0	7.2	9.6
	FEBRUARY *	-	7.7	7.7	6.0	−23.0
	MARCH *	-	6.6	6.6	4.6	−31.3
	APRIL	6.4	6.4	6.4	2.7	−57.6
	MAY	6.4	6.6	6.5	3.1	−52.5
	JUNE	6.2	5.6	5.9	3.2	−44.9
	JULY	6.4	6.3	6.3	4.5	−28.9
	AUGUST	6.3	5.7	6.0	4.2	−30.5
	SEPTEMBER	6.1	6.2	6.1	4.0	−34.6
	OCTOBER	6.5	6.7	6.6	3.9	−41.2
	NOVEMBER	6.7	6.5	6.6	3.8	−42.3
	DECEMBER	6.2	6.2	6.2	3.3	−47.4

* The antibiotic prescribing as DID for these months was only obtained for the years of 2019 and 2020.

**Table 2:** Interrupted segmented regression time series analysis of antibiotics prescribing as DID in outpatient care.

	Time: January 2018 to March 2021		Immediate Effect		Long Term Effect	
	B	95%CI	B	95%CI	B	95%CI
Antibiotic prescribing (DID)	−0.009	(−0.043; 0.024)	−1.6 *	(−2.5; −0.70)	0.011	(−0.094; 0.12)
Third-generation cephalosporins prescribing (DID)	8.02 × 10^−5^	(−2.5 × 10^−4^; 4.1 × 10^−4^)	−0.010 *	(−0.019; −0.002)	1.5 × 10^−4^	(−0.001; 0.001)
Fluoroquinolone prescribing (DID)	−0.012 *	(−0.017; −0.008)	−0.24 *	(−0.37; −0.11)	0.005	(−0.010; 0.020)
Clarithromycin prescribing (DID)	−0.003	(−0.006; 0.001)	−0.18 *	(−0.27; −0.095)	0.006	(−0.002; 0.015)

* *p* < 0.05. DID: defined daily dose per 1000 inhabitants per day; B: non-standardized coefficient; CI: confidence interval.

**Table 3:** Interrupted segmented regression time series analysis of the Watch antibiotics group prescribing as DID in outpatient care.

	Time: April 2018 to March 2021		Immediate Effect		Long Term Effect	
	B	95%CI	B	95%CI	B	95%CI
Watch antibiotics group prescribing (DID)	−0.020	(−0.056; 0.016)	−2.2 *	(−3.0; −1.3)	−0.043	(−0.13; 0.046)

* *p* < 0.05. DID: defined daily dose per 1000 inhabitants per day; B: non-standardized coefficient; CI: confidence interval.

## Data Availability

Publicly available datasets were analyzed in this study. This data can be found here: https://bicsp.min-saude.pt/pt/investigacao/Paginas/medicamentoprescritor_publico.aspx?isdlg=1.

## Supplementary Materials

The following are available online at www.mdpi.com/article/10.3390/antibiotics10091040/s1, Figure S1: Monthly prescribing trend of oral antidiabetics as DID prescribed in outpatient care. Blue line (−): tendency line, after model adjustment; Blue dashed line: expected tendency line with no COVID-19 emergence; Red dashed line: Covid-19 emergence (March 2020); +: oral antidiabetic monthly prescription in DID, Table S1: Monthly comparison analysis of the prescription of several antibiotic groups and clarithromycin as DID in outpatient care, before and after COVID-19 emergence; Figure S1: Monthly prescribing trend of oral antidiabetics as defined daily dose per 1000 inhabitants per day (DID) prescribed in outpatient care; Table S2: Interrupted segmented regression time series analysis of oral antidiabetics’ prescribing as DID in outpatient care.
