# Supplementary material for: The Impact of the COVID-19 Pandemic on Antibiotic Prescribing Trends in Outpatient Care: A Nationwide, Quasi-Experimental Approach
Source: Antibiotics (Basel). 2021 Aug 25;10(9):1040. doi: 10.3390/antibiotics10091040 (PMC8465975; doi:10.3390/antibiotics10091040)
Supplement: Supplementary file 1 [file antibiotics-10-01040-s001.zip › antibiotics-1305339-supplementary.pdf]

Supplementary Materials

The impact of the COVID-19 pandemic on antibiotic prescribing trends in outpatient care: a nationwide, quasi-experimental approach

Table S1. Monthly comparison analysis of the prescription of several antibiotic groups and clarithromycin as defined daily dose per 1000 inhabitants per day (DID) in outpatient care, before and after COVID-19 emergence.

		2018	2019	Mean 2018 and 2019	2020	Change in Percentage
3rd generation Cephalosporins prescribing (DID)	JANUARY	0.11	0.10	0.10	0.11	5.4
	FEBRUARY	0.11	0.10	0.10	0.10	−2.8
	MARCH	0.090	0.085	0.09	0.07	−19.9
	APRIL	0.085	0.080	0.08	0.06	−31.6
	MAY	0.078	0.082	0.08	0.06	−25.9
	JUNE	0.082	0.076	0.08	0.07	−16.6
	JULY	0.076	0.079	0.08	0.08	−0.5
	AUGUST	0.077	0.073	0.08	0.08	2.1
	SEPTEMBER	0.078	0.078	0.08	0.07	−3.6
	OCTOBER	0.080	0.088	0.08	0.08	−8.9
	NOVEMBER	0.076	0.088	0.08	0.07	−9.7
	DECEMBER	0.079	0.10	0.09	0.07	−21.1
Fluoroquinolone prescribing (DID)	JANUARY	1.3	1.2	1.3	1.1	−15.8
	FEBRUARY	1.3	1.2	1.2	0.90	−27.8
	MARCH	1.2	1.1	1.2	0.81	−30.4
	APRIL	1.2	1.1	1.1	0.55	−51.4
	MAY	1.1	1.1	1.1	0.59	−46.9
	JUNE	1.1	0.96	1.0	0.62	−40.4
	JULY	1.2	1.0	1.1	0.78	−29.2
	AUGUST	1.0	0.92	0.98	0.71	−27.5
	SEPTEMBER	1.1	0.98	1.0	0.70	−31.7
	OCTOBER	1.1	1.1	1.1	0.67	−39.9
	NOVEMBER	1.1	1.1	1.1	0.65	−39.5
	DECEMBER	0.98	0.95	0.96	0.58	−39.8
Clarithromycin prescribing (DID)	JANUARY	1.1	1.0	1.1	0.87	−19.2
	FEBRUARY	1.0	0.97	1.0	0.78	−22.5
	MARCH	0.85	0.75	0.80	0.55	−31.5
	APRIL	0.73	0.68	0.71	0.21	−70.4
	MAY	0.72	0.68	0.70	0.20	−71.9
	JUNE	0.68	0.58	0.63	0.24	−62.3
	JULY	0.61	0.59	0.60	0.30	−49.6
	AUGUST	0.56	0.51	0.54	0.30	−44.5

SEPTEMBER	0.57	0.57	0.57	0.34	−40.5
OCTOBER	0.66	0.67	0.66	0.35	−46.5
NOVEMBER	0.73	0.63	0.68	0.34	−49.6
DECEMBER	0.70	0.68	0.69	0.29	−57.8

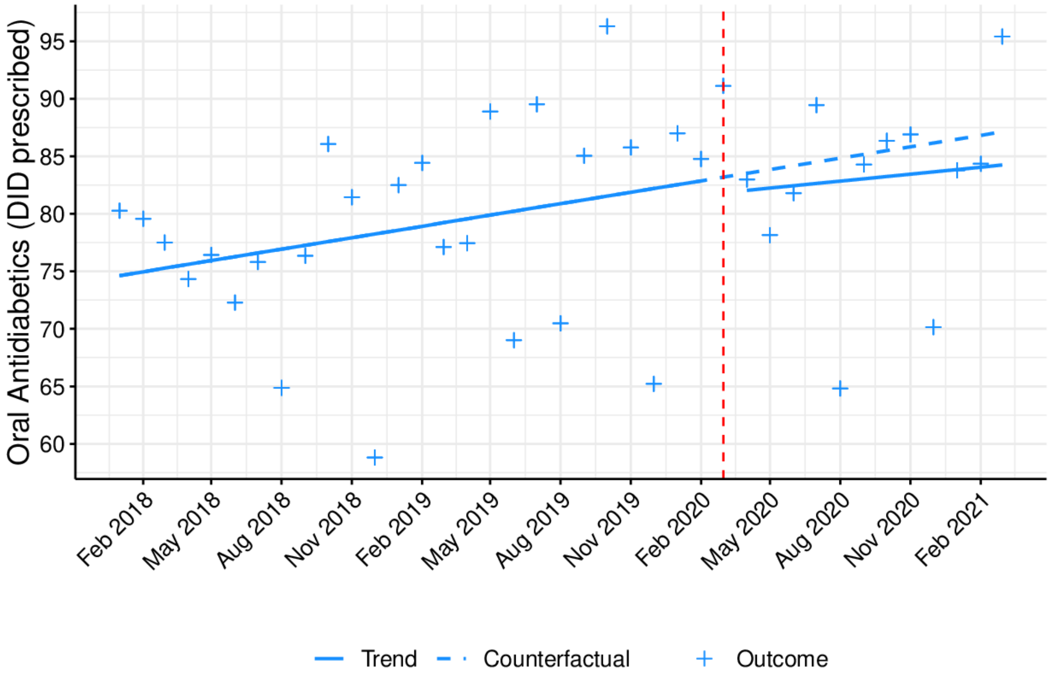

Figure S1. Monthly prescribing trend of oral antidiabetics as DID prescribed in outpatient care. Blue line (−): tendency line, after model adjustment; Blue dashed line: expected tendency line with no COVID-19 emergence; Red dashed line: Covid-19 emergence (March 2020); **+**: oral antidiabetic monthly prescription in DID.

Table S2. Interrupted segmented regression time series analysis of oral antidiabetics' prescribing as DID in outpatient care.

	Time: January 2018 to March 2021		Immediate Effect		Long Term Effect	
	B	95%CI	B	95%CI	B	95%CI
Oral Antidiabetic prescribing (DID)	0.67 *	[0.16; 1.2]	0.41	[−11.4; 12.2]	−0.79	[−2.2; −0.59]

* $p < 0.05$. DID: Defined Daily Dose per 1000 inhabitants per day; B: non-standardized coefficient; CI: Confidence Interval.
